# Hyperhomocysteinemia‐Driven Ischemic Stroke: Unraveling Molecular Mechanisms and Therapeutic Horizons

**DOI:** 10.1002/fsn3.70517

**Published:** 2025-07-03

**Authors:** Bin Li, Yushun Kou, Lingna Zhang, Lin Yi

**Affiliations:** ^1^ School of Traditional Chinese and Western Medicine Gansu University of Chinese Medicine Lanzhou China

**Keywords:** folate, homocysteine, hyperhomocysteinemia, ischemic stroke, vitamin

## Abstract

Homocysteine is a toxic intermediate in the metabolism of methionine, and impaired homocysteine metabolism can lead to hyperhomocysteinemia, whose clinical relevance to ischemic stroke has been confirmed by many studies and is considered an independent risk factor for ischemic stroke. This article reviews and analyzes studies related to ischemic stroke due to hyperhomocysteinemia. Firstly, the mechanism of hyperhomocysteinemia was examined, and it was clarified that hyperhomocysteinemia is the result of mutations in the genes of key enzymes involved in homocysteine metabolism or nutritional disorders of cofactors involved in homocysteine metabolism. Secondly, we reviewed that hyperhomocysteinemia may lead to a series of pathological processes such as vascular injury, thrombosis, and vasoconstriction through the mechanisms of inflammation and oxidative stress, neurotoxicity, and epigenetic dysregulation, which ultimately lead to the development of ischemic stroke. The article also provides a review of research on the prevention and treatment of ischemic stroke associated with hyperhomocysteinemia, specifically describing three large clinical vitamin supplementation trials, which, despite the heterogeneity of findings, provide partial evidence for prevention. This review will provide some insights and thoughts for studying the biological mechanisms of hyperhomocysteinemia‐associated ischemic stroke and exploring novel therapies for its prevention and treatment.

## Graphical abstract

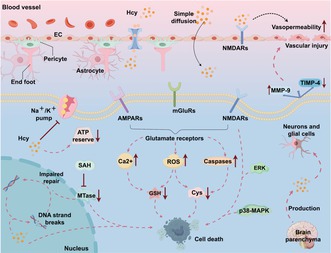

## 1 Introduction

Stroke is a serious threat to health and a leading cause of disability and death worldwide (Donnan et al. 2008). The most common type of stroke is ischemic stroke (IS), which accounts for 87% of all strokes (Qiu and Xu 2020). According to the World Health Organization, about 15 million people are diagnosed with IS each year, and about 10 million die or become permanently disabled (Ziganshina et al. 2023). Since stroke can lead to long‐term disability, early detection of risk factors and active control are necessary for effective stroke prevention.

Homocysteine (Hcy) is an amino acid that is produced by the metabolism of methionine (Met), which is normally converted in the body to other compounds such as cysteine or Met. A disorder in its metabolism results in elevated levels of Hcy in the body, a condition known as hyperhomocysteinemia (HHcy). Serum Hcy levels between 5 and 15 μmol/L are considered normal, whereas HHcy is defined as blood Hcy levels higher than 15 μmol/L, with values between 16 and 30 μmol/L being mild, between 31 and 100 μmol/L being moderate, and more than 100 μmol/L being severe (Kang et al. 1992). The prevalence of HHcy in China is 27.5%, which is significantly higher than that in developed countries (Yang et al. 2014).

Hcy has received much attention in recent years in the study of neurological disorders, especially in IS (Rabelo et al. 2022). Several epidemiologic studies have shown that elevated levels of Hcy are closely associated with the processes of IS onset (Liu et al. 2021), progression (Herrmann and Obeid 2011), recurrence (Wang et al. 2023), and prognosis (Ma et al. 2021), and thus Hcy is considered an independent risk factor for IS (Ma et al. 2021). However, some studies have shown that mildly elevated Hcy levels do not increase the risk of IS (Sacco et al. 2004). Thus, suggesting uncertainty in the relationship between Hcy and IS. Meanwhile, several clinical trials have explored the therapeutic effects of folate, vitamin B6 (VB_6_), and vitamin B12 (VB_12_) being used to reduce Hcy levels (Toole et al. 2004; Saposnik et al. 2009; Vitatops Trial Study Group 2010). Some studies have suggested that this intervention may have some benefit in reducing the occurrence of IS (Saposnik et al. 2009); however, others have shown that lowering Hcy with high‐dose vitamin therapy does not reduce the risk of IS recurrence in IS patients with mildly elevated Hcy levels (Toole et al. 2004); it has also been shown that daily administration of folate, VB_6_, and VB_12_ does not appear to be more effective than placebo in reducing the incidence of IS (Vitatops Trial Study Group 2010). This suggests that the results of studies of folate and vitamin therapy to reduce Hcy levels and thereby prevent the onset and progression of IS are not yet fully consistent.

For these reasons, this article reviews the possible reasons for the occurrence of HHcy, the potential mechanisms of HHcy‐associated IS, and the pathological basis of HHcy‐associated IS, and summarizes the current treatments for HHcy‐associated IS to provide ideas for the study of HHcy‐associated IS, as well as recommendations for the prevention and treatment of HHcy‐associated IS.

## 2 Mechanisms of HHcy

**FIGURE 1:**
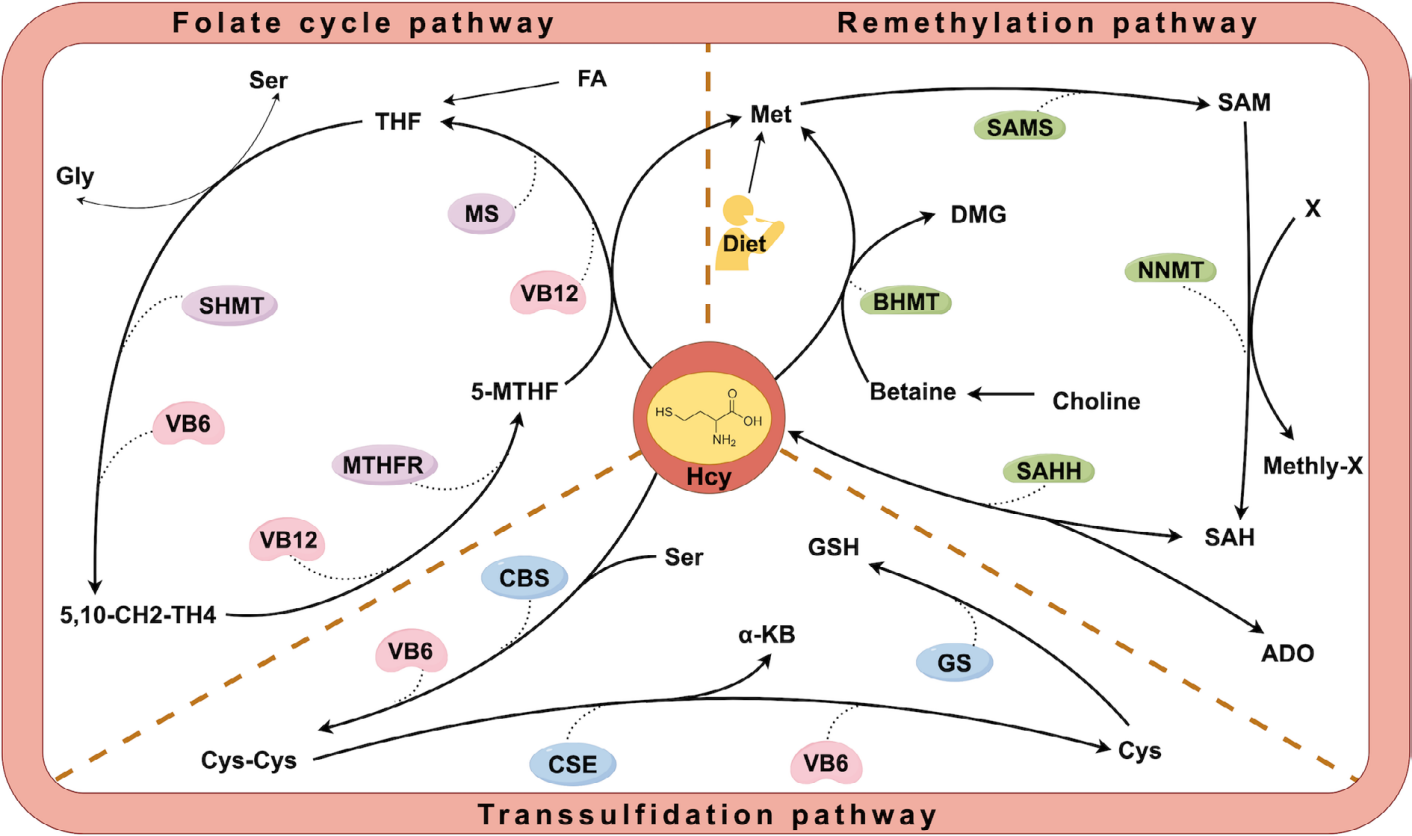
Homocysteine metabolism. The association among the three major pathways of remethylation, transsulfuration, and folate cycle in homocysteine metabolism. The folate cycle generates 5‐MTHF, which promotes methylation of methionine; the transsulfuration pathway converts homocysteine into glutathione. It emphasizes the crucial role of auxiliary factors such as vitamin B6 and B12 in maintaining metabolic balance.

There are two main pathways for the metabolism of Hcy (Figure 1): (i) the remethylation pathway (Piazzolla et al. 2019), which consists of a folate‐dependent pathway and a folate‐independent pathway. Folate‐dependent pathway: Hcy forms Met and tetrahydrofolate (THF) in the presence of VB_12_‐dependent methionine synthase (MS) and 5‐methyltetrahydrofolate (5‐MTHF) provides methyl for this reaction; Folate‐independent pathway: Hcy and betaine produce Met and dimethylglycine (DMG) by the action of betaine homocysteine methyltransferase (BHMT). The generated Met is then converted to S‐adenosylmethionine (SAM) by S‐adenosylmethionine synthase (SAMS), which generates S‐adenosylhomocysteine (SAH) after transferring the methyl group to the substrate by nicotinamide‐N‐methyltransferase (NNMT), which generates Hcy and adenosine (ADO) by the action of S‐adenosylhomocysteine hydrolase (SAHH). (ii) The transsulfuration pathway (Hadithi et al. 2009): in the presence of VB_6_ as a cofactor, Hcy undergoes a transsulfuration reaction via cystathionine‐β‐synthase (CBS) catalyzing serine (Ser) to form cystathionine (Cys‐Cys), which is then converted to cysteine (Cys) and α‐keto butyric acid (α‐KB) by cystathionine‐γ‐ lyase (CSE); Cys can be generated into glutathione (GSH) by glutathione synthase (GS).

Based on the metabolic pathway of Hcy, we can find that HHcy may be caused by a deficiency of enzymes and vitamin cofactors involved in Hcy metabolism (Ostrakhovitch and Tabibzadeh 2019). In addition to this, excess intracellular Hcy can also contribute to HHcy by being released from the intracellular to the extracellular fluid through the intra‐ and extracellular concentration difference and then transported to the systemic circulation via the bloodstream, a process that is a self‐protective mechanism of the cell to prevent the accumulation of Hcy within the cell (Schalinske and Smazal 2012).

### 2.1 Cofactor Nutritional Disorders

Nutrition is a modifiable risk factor for IS, and as people age, their ability to absorb certain nutrients decreases, VB_12_ being a prime example (Spence 2019). Elderly people deficient in VB_12_ are at higher risk of IS and have a worse prognosis for IS (Yahn et al. 2021). In the Chinese population, HHcy prevalence may be more associated with inadequate intake of Hcy metabolizing cofactors such as vitamins (Yang et al. 2014).

Zhou et al. (2023) evaluated the relationship between IS and serum VB_12_ status in 2212 hospitalized patients. It was found that low levels of VB_12_ predicted the risk of IS in hospitalized patients, and early and timely VB_12_ supplementation improved the short‐term prognosis of IS patients. Interestingly, this study also found that IS patients aged 70 years or older had higher folate concentrations and lower Hcy concentrations compared to non‐IS patients. It has been shown that “excess folate” occurs at low VB_12_ concentrations (Bailey et al. 2020), which may indicate that patients with high folate concentrations may have problems with metabolism and excretion, that is, decreased uptake by the liver or kidneys or increased release from the cells in the elderly (Samson et al. 2022). This is because it has been shown that end‐stage renal disease affects the ability to transsulfurize Hcy, which can ultimately lead to increased plasma concentrations of Hcy (Long and Nie 2016).

Mbs et al. (2024) placed 10‐month‐old male and female mice in either a VB_12_‐deficient diet group or a control group, and measurements of motor function were taken before and 4 weeks after IS, and mouse tissues were collected to assess potential mechanisms. It was found that after IS, motor function was impaired in VB_12_‐deficient mice compared with controls; Hcy levels were elevated in both plasma and liver tissues; and, in addition, choline metabolites were altered in ischemic brain tissues as a result of VB_12_ deficiency. The study confirms that VB_12_ deficiency worsens the prognosis of IS in mice, and the mechanism leading to this change may be a result of Hcy‐induced neuronal damage and compensation of choline metabolism in brain tissue.

### 2.2 Mutations in Key Enzyme Genes

Some genetic factors, such as mutations in genes for key enzymes involved in the Hcy metabolism pathway, may affect the metabolism of Hcy, leading to elevated plasma levels of Hcy, which are associated with an increased risk of IS.

Methylenetetrahydrofolate reductase (MTHFR) is an important enzyme involved in Hcy metabolism that stimulates the conversion of 5,10‐methylenetetrahydrofolate (5,10‐CH2‐TH4) to 5‐MTHF, which is a co‐substrate for the remethylation of Hcy to Met (Selhub 1999). The MTHFR gene is located on chromosome 1 (1p36.3), and it encodes a 77 kDa dimeric protein that is the rate‐limiting enzyme in the methyl cycle (Goyette et al. 1998). Abato et al. (2022) performed photothrombotic (PT) injury in 3‐month‐old male Mthfr^+/−^ and wild‐type littermates. It was found that Mthfr^+/−^ mice used their undamaged forepaws more when exploring the cylinder and had a larger stroke volume than wild‐type littermates; in brain tissue of Mthfr^+/−^ mice, methionine adenylyltransferase IIα (MAT2A) protein levels were reduced in the injured hemisphere, whereas hypoxia‐inducible factor 1α (HIF‐1α) levels were increased in the non‐injured hemisphere; Enhanced antioxidant responses at the injury site were manifested by increased levels of nuclear factor erythroid 2‐related factor 2 (Nrf2) and superoxide dismutase 2 (SOD2). This animal study shows that Mthfr^+/−^ mice are more susceptible to PT‐induced stroke injury by modulating cellular responses.

The MTHFR gene has at least two functional polymorphisms, namely, A1298C (rs 1,801,131), in which the A on base pair 1298 is replaced by a C, resulting in the substitution of glutamic acid for alanine at position 429 in the amino acid sequence of the MTHFR that it encodes, with three genotypes, AA, AC, and CC; and C677T (rs 1801133), where the C on base pair 677 is replaced by a T, resulting in the substitution of an alanine for valine at position 222 in the amino acid sequence of the encoded MTHFR, with three genotypes, CC, CT, and TT (Botto and Yang 2000). A previous study in an Arab population showed that the prevalence of the heterozygous C677T genotype was 25.8% in healthy individuals, while the prevalence of the heterozygous A1298C genotype was 51.5% (Abu‐Amero et al. 2003). The C677T polymorphism in the MTHFR gene reduces the thermal stability of the MTHFR enzyme, and the MTHFR enzyme under this homozygous polymorphism is 50%–60% less active at 37°C compared to normal non‐mutated controls (Kang et al. 1988). A study by Mazdeh et al. (2021) evaluated the association between C677T and A1298C and IS risk in an Iranian population and found that C677T polymorphism resulted in nearly 70% and 35% reduction in MTHFR activity in TT and CT genotypes, respectively. Huang et al. (2022) investigated whether the C677T gene polymorphism of MTHFR was associated with IS risk and Hcy levels in a Chinese population. It was found that the C677T gene polymorphism of MTHFR affects plasma Hcy levels, with the TT genotype having the highest plasma Hcy levels; the TT genotype has a higher IS risk than the CC genotype; and the T allele is a susceptibility allele for IS. Ames et al. (2022) compared the age of first IS in 82 adolescent patients with the TT genotype, 54 with the TC genotype, and 34 with the CC genotype of MTHFR, assessed MTHFR gene polymorphisms, and determined the predictors of the type of cerebrovascular involvement leading to IS (small‐vessel disease (SVD) versus large‐vessel disease (LVD)). Adolescents with the TT genotype were found to have their first occlusion an average of 5 years earlier compared with the CC genotype; Hcy predicted IS secondary to SVD. These experiments have demonstrated that the two‐heterozygous state of MTHFR, especially C677T, leads to decreased MTHFR activity, which in turn leads to impaired Hcy metabolism, elevated levels, and increased risk of IS (Frosst et al. 1995).

It has been shown that the A/G gene polymorphism of NNMT (rs 694539) is also associated with HHcy (Souto et al. 2005). NNMT is an enzyme involved in the synthesis of SAH; it utilizes the methyl group produced during the conversion of SAM to SAH and breaks down nicotinamide and other pyridine compounds in the reaction (Aksoy et al. 1994). It is noteworthy, however, that in a Japanese study, there was no difference in plasma Hcy concentrations between the AA, AG, and GG genotypes of NNMT; however, the GG genotype of NNMT was associated with elevated plasma Hcy only when it coexisted with other confounding factors, such as age, folate deficiency, and/or polymorphisms of the C677T gene in MTHFR (Zhang et al. 2007).

The gene for Methionine synthase reductase (MTRR) is a housekeeping gene located on chromosome 5 (p15.2–p15.3; Rai et al. 2012). Although MTRR is not directly involved in the conversion of Hcy to Met, it maintains MS activity and is, therefore, a key enzyme in Hcy metabolism. Variants in the gene encoding MTRR may reduce MTRR activity, and reduced MTRR activity leads to increased Hcy concentrations, which have been associated with several health risks, including cardiovascular disease (Basir 2019). The MTRR gene has a functional polymorphism, that is, A66G (rs1801394), in which the A on base pair 66 is replaced by a G, resulting in the substitution of isoleucine by a Met at position 22 in the amino acid sequence of the MTRR it encodes, with three genotypes, AA, AG, and GG (Olteanu and Banerjee 2001). Li, Zhao, et al. (2020) investigated the association of MTRR gene variants and promoter methylation with IS in Chinese HHcy patients by analyzing the A66G polymorphism and promoter methylation of MTRR in the HHcy population with and without IS in Zhengzhou City, Henan Province, China. The A66G gene polymorphism of MTRR and elevated plasma Hcy levels were found to be significantly associated with an increased risk of IS in HHcy patients, and it was further concluded that the G allele of the A66G polymorphism of MTRR appeared to be susceptible to IS in the Chinese HHcy population.

CBS deficiency affects the conversion of Hcy to Cys‐Cys, leading to the accumulation of Hcy (Morris et al. 2017). A meta‐analysis (Ding et al. 2012) provided evidence that CBS T833C gene polymorphisms were associated with stroke risk; however, the results differed between the Chinese and Caucasian subgroups, and in the Chinese subgroup, the results showed that CBS T833C gene polymorphisms led to an increased incidence of stroke. The mutation frequency of MS A2756G plays an important role in the synthesis and metabolism of Hcy, which is significantly lower in the Chinese population than in Caucasians (Zhang et al. 2020).

## 3 Potential Mechanisms of HHcy‐Related IS

Chronically elevated levels of Hcy have been implicated in the pathogenesis of IS because Hcy triggers a complex process that includes a series of neurotoxicity, including increased inflammation and oxidative stress, reactive oxygen species generation, membrane lipid and protein oxidation, protein homocysteination, Ca2+ dysregulation, and DNA methylation, and these events, in conjunction with epigenetic risk factors, can lead to neuronal apoptosis and dysregulation of the blood–brain barrier (BBB) which ultimately leads to the development of IS (Lehotský et al. 2016).

### 3.1 Inflammation and Oxidative Stress

Hcy can promote oxidative stress in neural and/or vascular tissues, increase the concentration of inflammatory cytokines to enhance the inflammatory response, which in turn leads to cellular damage in various tissues, and can promote the formation of atherosclerosis (AS) and rupture of AS plaques, which are all closely related to the development of IS (Djuric et al. 2018).

Elevated Hcy levels produce cytotoxic and pro‐inflammatory effects and ultimately lead to a series of pathological processes such as vascular endothelial dysfunction, lipid metabolism disorders, and remodeling of vascular tissues, which can lead to thrombosis and AS (Boldyrev et al. 2013). It has been shown that elevated Hcy stimulates pro‐inflammatory pathways in vascular cells, leading to recruitment of leukocytes to the vessel wall via the expression of adhesion molecules on endothelial cells (EC) and circulating monocytes and neutrophils, infiltration of leukocytes into the arterial wall via increased secretion of chemokines, as well as the differentiation of monocytes into cholesterol‐scavenging macrophages; in addition, Hcy stimulates proliferation of vascular smooth muscle cells and subsequent production of extracellular matrix (Papatheodorou and Weiss 2007). The study suggests to us that elevated Hcy promotes these inflammatory response events and leads to the formation of AS lesions. Zhang et al. (2021) used Wistar–Kyoto rats to study the immunomodulatory mechanism of HHcy on brain injury. The results showed significant suppression of regulatory T‐cell (Treg) response effects and an upregulation of inflammatory response effects of T‐helper cell 17 (Th17) in the Met group compared to the treatment group (with VB6, VB12, and folate). The expression levels of IL‐17A and retinoic acid‐associated orphan receptor γ t were significantly up‐regulated in the Met group compared with the treatment group, whereas the mRNA levels of forkhead box P3 were significantly downregulated in the Met group. In conclusion, the present study demonstrated that HHcy can promote inflammatory response by triggering Treg/Th17 immune imbalance, which can aggravate brain tissue injury. Dietary interventions (including VB6, VB12, and folate) cannot only reduce the level of HHcy in the blood but also reduce the inflammatory response and correct the Treg/Th17 immune imbalance, which can ameliorate brain tissue injury.

Another pathogenic process associated with Hcy‐induced inflammatory responses may involve modification of protein structure. Protein homocysteinylation consists of (i) S‐homocysteinylation and (ii) N‐homocysteinylation, both of which are considered post‐translational protein modifications, and it has been demonstrated that proteins modified by N‐homocysteinylation act as neoantigens, triggering the activation of inflammatory responses that are a key component of the etiology of AS and stroke (H. Jakubowski 2011).

Experiments using genetic or diet‐induced HHcy animal models or EC cultured under conditions of high Hcy levels have shown that the accumulation of reactive oxygen species (ROS), especially superoxide anion, is increased (Mohamed et al. 2017). Hcy‐induced oxidative stress may be due to decreased expression and/or activity of key antioxidant enzymes and increased enzymes that generate ROS, both of which work together to cause an imbalance in the oxidative‐antioxidant system, where ROS include superoxide anion, hydroxyl radicals, persulfate, hydrogen peroxide or other peroxides, hypochlorite and its organic analogs (Wang et al. 2018).

Lehotský et al. (2016) classified the mechanisms of Hcy‐induced oxidative stress into the following categories: (i) limited activity of cellular antioxidant enzymes, (ii) Hcy auto‐oxidation, (iii) generation of superoxide anion by nitric oxide synthase (NOS) via uncoupling of endothelial NOS (eNOS), (iv) disruption of extracellular superoxide dismutase on endothelial surfaces, and (v) activation of NADPH oxidase.

In the vascular system, moderate concentrations of ROS act as signaling molecules and play an important role in regulating various vascular cell functions. They are involved in the regulation of vascular tone, oxygen sensing, cell growth and proliferation, apoptosis, and inflammatory responses (Zhang and Gutterman 2007). In the context of atherosclerosis and inflammation, ROS control and regulate cytokine, chemokine, and adhesion molecule genes, particularly through redox signaling, which may promote intravascular thrombosis (Lavrovsky et al. 2000). ROS may also be toxic to cells and tissues by promoting lipid peroxidation of membranes (loss of membrane function and increased permeability) and by generating lipid autoperoxidation through oxidative damage to LDL, DNA damage leading to mutation and death, and cross‐linking or sulphation of sulfhydryl‐rich proteins (leading to stiff aging proteins, especially collagen in the extracellular matrix; Hayden and Tyagi 2002).

Ozkul et al. (2007) measured serum nitric oxide (NO), malondialdehyde (MDA), and glutathione (GSH) levels in 70 stroke patients 48 h after stroke and found that acute stroke patients had significantly higher serum NO, MDA, and GSH levels. In addition, Hcy can be converted to cystathionine by CBS, and the effect of CBS may increase the level of the antioxidant GSH, which may be a compensatory mechanism to counteract the potential oxidative damage caused by increased Hcy (Mosharov et al. 2000).

Elevated levels of Hcy lead to increased cellular oxidative stress, which is also increased in the mitochondria. Studies have shown that Hcy promotes mitochondrial damage and alters mitochondrial gene expression and function, suggesting that mitochondrial oxidative stress is involved in the development of IS (Austin et al. 1998). It was found that in human megakaryocytic leukemia cells, Hcy‐induced the expression of the mitochondrial electron transport chain genes cytochrome c oxidase III/ATPase 6 and 8 in a concentration‐ and time‐dependent manner. In contrast, in the presence of Cu2+, which is known to produce hydrogen peroxide, Hcy significantly reduces mitochondrial RNA levels of cytochrome c oxidase III/ATPase 6 and 8, leading to severe morphological changes in mitochondrial ultrastructure and inhibition of cell growth and mitochondrial respiration rates (Perez‐ De‐Arce et al. 2005).

Wu et al. (2019) found that Hcy induces endoplasmic reticulum oxidoreductase 1α (ERO1A) expression and produces endoplasmic reticulum (ER) stress and inflammation in human umbilical vein endothelial cells (HUVEC) and arteries of HHcy mice. The specific mechanism is that Hcy upregulates ERO1A expression by promoting the binding of hypoxia‐inducible factor 1α to the ERO1A promoter, but it is noteworthy that Hcy significantly increases the ratio of GSH/GSSG in the ER, and thus can ectopically activate ERO1A to produce H_2_O_2_ and trigger ER oxidative stress. In contrast, the antioxidant pathway mediated by endoplasmic reticulum glutathione peroxidase 7 (GPx7) was downregulated in HHcy mice. Further studies revealed that ERO1A knockdown and GPx7 overexpression protected EC from HHcy‐induced ER oxidative stress and inflammation. This study suggests that targeting ER redox homeostasis can be used to intervene in HHcy‐related vascular diseases; the findings also highlight the importance of ER oxidative stress in HHcy‐induced vascular endothelial inflammation and reveal the possibility of targeting ER redox homeostasis to intervene in endothelial dysfunction‐associated vascular diseases, including AS, IS, and ischemia/reperfusion injury.

It is well known that high‐density lipoprotein (HDL) transports lipids from the blood through the bloodstream to the liver and exerts an anti‐AS effect. In a multivariate Mendelian randomization study, increased HDL was found to be associated with a reduced risk of small vessel occlusion (SVO)‐type stroke and was a protective factor for IS (Hindy et al. 2018). A study by Kim et al. (2020) showed that elevated Hcy levels during AIS were strongly associated with lower total cholesterol, lower low‐density lipoprotein (LDL), and lower HDL. However, according to stroke subtypes, only SVO stroke patients had elevated Hcy levels that were significantly associated with lower HDL levels. The results of the present study suggest that Hcy may play a mediating role between high HDL and the occurrence of SVO‐type stroke, but the reasons for the association of Hcy with HDL in patients with AIS remain to be determined. Furthermore, it has been shown that Hcy oxidizes LDL, produces thiolated LDL, inhibits intracellular antioxidant enzymes, and reduces NO bioavailability (Komatsu et al. 2016). Based on the hints from the above two studies, we conjecture whether Hcy causes HDL oxidation and thus plays a mediating role between HDL and SVO‐type stroke occurrence. However, this requires future studies to clarify the role of Hcy on lipid metabolism during stroke onset.

### 3.2 Mechanisms of Neurotoxicity

**FIGURE 2:**
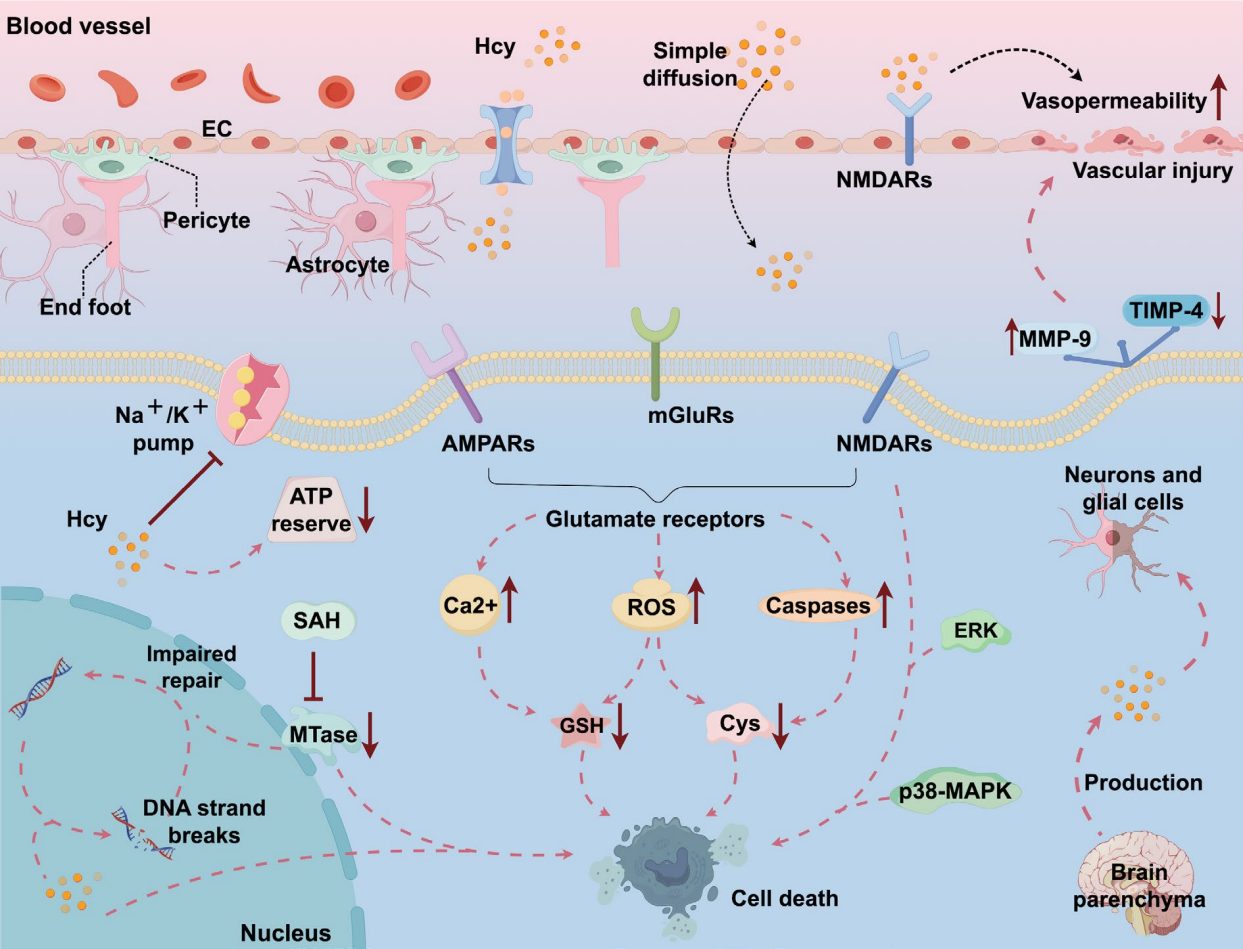
Neurotoxicity of Hcy. (A figure that best represents the scope of the paper.). Homocysteine can reduce the tightness of endothelial cell connections, increase vascular permeability, and damage the blood–brain barrier. Homocysteine activates various glutamate receptors on the surfaces of neurons and glial cells, triggering excitotoxicity, promoting the production of ROS, disrupting the antioxidant system, and leading to DNA damage and cell apoptosis.

Regarding how Hcy accumulates in the brain, some animal studies have shown that in addition to simple diffusion with a concentration gradient, Hcy can be transported via BBB epithelial cells via specific saturable receptors (Grieve et al. 1992). It has also been shown that Hcy production is increased in neuronal cells cultured in folate‐deficient media, suggesting that Hcy can be produced in the brain parenchyma (Ashline et al. 2003). This is because although all tissues are capable of producing Hcy, the transsulfuration pathway of Hcy occurs only in the liver and kidney, whereas tissues such as the blood vessels and the brain utilize the remethylation pathway as the only option, and therefore, as the activity of enzymes such as MTHFR decreases significantly in tissues such as the blood vessels and the brain, Hcy is not able to be remethylated to Met and therefore accumulates in the bloodstream and the nervous system (Petras et al. 2014; Figure 2).

It is well known that the process of IS is very complex and involves multiple pathophysiologic mechanisms. There is evidence that excitotoxic mechanisms may play a role in Hcy‐induced neurotoxicity (Deb et al. 2010). Overstimulation of neuronal excitatory glutamate receptors can lead to higher levels of cytoplasmic calcium and free radicals in neurons, as well as cause aberrant protein expression, leading to Cys and GSH dysfunction, and reduced brain tissue resistance to oxidative stress and tissue repair (Han et al. 2022).

The N‐methyl‐D‐aspartate (NMDA) receptor is an ionotropic glutamate receptor that mediates messaging in most excitatory neuronal synapses, and it is widely accepted that overactivation of NMDA receptors mediates neuronal damage after ischemia (Simon et al. 1984). On the one hand, it has been shown that Hcy directly binds to and activates neuronal NMDA receptors, leading to cell death (Lipton et al. 1997) due to the transient activation of extracellular regulated protein kinase (ERK) and p38 mitogen‐activated protein kinase (MAPK) (Poddar and Paul 2013), which is different from the downstream signaling pathways triggered by other NMDA receptor agonists. On the other hand, it has also been shown that mild elevation of Hcy directly activates NMDA receptors on cerebrovascular EC, leading to disruption of the BBB and an increase in permeability, which is normally used by the cerebrovascular endothelium to limit the influx of potentially neurotoxic compounds between the circulating blood and the central nervous system (Beard et al. 2011). Kim and Pae (1996) demonstrated the role of NMDA receptors in the mechanism of Hcy‐induced neurotoxicity in in vitro experiments using cultured neurons. In a genetic model of CBS‐deficient mice, experiments by Gu et al. (2020) confirmed that Cbs^+/−^ mice showed elevated plasma Hcy and that elevated plasma Hcy exacerbated cerebral vascular injury, whereas administration of the NMDA receptor antagonist, memantine, protected Cbs^+/−^ mice from stroke and had a protective effect on the BBB. This experiment demonstrates that elevated plasma Hcy exacerbates cerebrovascular injury and suggests that NMDA antagonists may be a strategy to prevent reperfusion injury after acute IS in patients with mild HHcy. Lipton et al. (1997) found that Hcy interacts with NMDA receptors in a complex manner, acting as both a partial agonist at the glutamate‐binding site and a partial antagonist at the glycine regulatory site. It has been demonstrated that the neurotoxic effects of Hcy are dependent on the presence of high concentrations of glycine; glycine is essential for the displacement of Hcy from its binding sites, which in turn is a prerequisite for the activation of NMDA channels. In conclusion, the mechanism by which HHcy affects and causes IS may lie in the fact that elevated levels of Hcy lead to enhanced excitatory glutamatergic neurotransmission in different brain regions, which results in excess Ca^2+^ influx and increased ROS generation, thereby inducing neuronal damage (Zieminska et al. 2006).

In addition, Hcy is an agonist for two other classes of glutamate receptors, (i) metabotropic (group I and III) receptors and (ii) ionotropic (AMPA) receptors (Boldyrev et al. 2013). Overstimulation of these receptors leads to elevated cytoplasmic Ca^2+^ levels, increased free radical production, and activation of caspases that lead to apoptosis (Mattson and Shea 2003). A study by Ziemińska et al. (2003) found that in cultured rat cerebellar granule neurons, in addition to NMDA receptors, group I metabotropic glutamate receptors (mGluRs) were also involved in Hcy‐induced neurotoxicity. For the first time, it was shown that Hcy‐induced neurotoxicity is mediated by group I mGluRs and NMDA receptors, but remarkably, this study found that this process was not accompanied by a massive influx of extracellular Ca^2+^ into neurons.

In addition to excitotoxic mechanisms, neurotoxic mechanisms of HHcy include disruption of methylation processes (Duan et al. 2002). Hcy is a cytotoxic sulfur‐containing amino acid that induces DNA strand breaks (Ho et al. 2002). In this process, intracellular accumulation of SAH inhibits several methyltransferases, ultimately leading to impaired repair of damaged DNA and apoptotic cell death (Ziemińska et al. 2003). In addition, Hcy activates the neuronal apoptotic program (Mattson and Shea 2003), inhibits the cell membrane sodium‐potassium pump (Folstein et al. 2007), induces neuronal dysfunction, and promotes the development of acute IS (Chen et al. 2022). Hcy induces DNA breaks in cultured neurons by a mechanism that may involve impaired transmethylation of DNA, and cultured neurons treated with Hcy eventually deplete their ATP reserves in an attempt to repair Hcy‐induced DNA damage (Kruman et al. 2000). Hcy has also been shown to negatively affect neural stem cell viability, proliferation, and differentiation through the promotion of autophagy, a mechanism that may contribute to decreased neural repair after stroke (Wang et al. 2019).

Another pathogenic process associated with Hcy‐induced neurotoxicity may involve modification of protein structure. Protein homocysteinylation includes (i) S‐homocysteinylation and (ii) N‐homocysteinylation, both of which are considered post‐translational protein modifications, and N‐homocysteinylated proteins on the luminal surface of vascular mature EC are recognized by specific antibodies, and the interaction of this neoantigen with auto‐antibodies results in the activation of circulating macrophages, which leads to recurrent damage to the vascular endothelium; In addition, Hcy‐thiolactone (HTL) impairs the self‐regenerative capacity of the vascular endothelium by directly inhibiting lysyl oxidase, which is responsible for the proper cross‐linking of collagen and elastin in the arterial wall (Raposo et al. 2004).

It has also been shown that high levels of Hcy exacerbate cortical neuronal cell damage after cerebral ischemia via oxidative damage‐mediated neuronal autophagy (Zhao et al. 2016). Tyagi et al. (2012) demonstrated homocysteinylated cytochrome‐c‐mediated autophagy in HHcy mice after cerebral ischemia, whereas cytochrome‐c translocates electrons in the system and facilitates bioenergetics, and the authors found that tetrahydro curcumin ameliorated autophagy during this condition by partially reducing homocysteinylation of cytochrome‐c through MMP‐9 activation. These studies suggest that Hcy‐mediated autophagy is a molecular mechanism of Hcy toxicity to neuronal cells during ischemic injury.

Not only are neurons affected by Hcy toxicity, but also glial cells, whose importance for brain homeostasis, assisting neurogenesis, determining the microstructure of gray matter, and energy metabolism has been well documented (Verkhratsky and Toescu 2006). Škovierová et al. (2015) evaluated the neurotoxicity of Hcy on glial cells using glioblastoma cell lines as a model system. They examined the viability of the cells by biochemical and cytological methods and found that significant cell death was observed when the concentration of Hcy in the culture medium was about 50 μmol/L. This suggests to us that Hcy‐induced impairment of neuronal function and damage to glial cells may be part of the pathogenesis of Hcy‐related IS.

### 3.3 Dysregulation of Epigenetic Mechanisms

The deleterious effects of Hcy also include the newly discovered dysregulation of epigenetic mechanisms. Studies have shown that epigenetic mechanisms such as DNA methylation, chromatin remodeling, RNA editing, noncoding RNAs, and microRNAs are also involved in the development of stroke (Kalani et al. 2014).

There is growing evidence that epigenetic inheritance, such as DNA methylation, plays an important role in the pathogenesis of IS and the pathophysiology of ischemic tolerance. DNA methylation is the transfer of methyl groups from activation donors, such as SAM, to cytosine residues by DNA Methyltransferase (DNMT) (Lund et al. 2004), thereby affecting the neuroprotective effects of ischemic tolerance (Hu et al. 2017). Blocking DNA methylation by 5′‐azacytidine or DNMT knockdown attenuates ischemic brain damage (Endres et al. 2000). Upregulation of methylation by DNA methylating agents can block the neuroprotective effects of ischemic tolerance (Maysami et al. 2008). It has been shown that methylases that regulate DNA methylation are implicated in these processes and that the aberrant metabolism of Hcy may affect the function of these methylases, which may lead to dysregulation of DNA methylation and silencing of gene function (Kalani et al. 2013).

Gou et al. (2021) aimed to determine whether high levels of Hcy exposure adversely affect brain hippocampal neural stem cell (NSCs) genesis using a rat model of transient focal cerebral ischemia; in addition, potential neurotoxicity mechanisms of Hcy were also investigated. Studies have shown that high levels of Hcy can lead to reduced levels of DNA methylation in ischemic brain tissue, inhibiting the self‐renewal ability of NSCs and ultimately inhibiting neurogenesis in ischemic brain tissue and that the induced DNA hypomethylation may be mainly caused by a reduction in the activity of DNMT, which is regulated by the concentration of SAM and SAH (Selhub 1999). The results of this study suggest that Hcy‐lowering interventions may help brain tissue maintain an appropriate DNA methylation state during neurogenesis in response to ischemic injury and may be a potential approach to treating stroke.

The gene methylation levels of key enzymes in the Hcy metabolic pathway are associated with the risk of IS, which may be because abnormal Hcy metabolism can lead to an imbalance in the concentrations of SAM and SAH, which affects DNMT activity and further influences the gene methylation levels of the key enzymes of editing (Selhub 1999).

Xu et al. (2020) found that hypermethylation of the MTHFR gene was a protective factor against IS, and high levels of MTHFR promoter methylation reduced the risk of IS in hypertensive patients, especially in men. Li et al. (2021) evaluated the value of methylation of five genes associated with Hcy metabolism [methylenetetrahydrofolate dehydrogenase 1 (MTHFD1), CBS, dihydrofolate reductase (DHFR), serine hydroxymethyltransferase 1 (SHMT1), and SAHH] for predicting the risk of developing IS in patients with hypertension in a population of hypertensive patients. Elevated levels of CBS, DHFR, and MTHFD1 methylation were found to be associated with reduced IS risk; furthermore, when CBS hypomethylation and DHFR hypomethylation were combined as biomarkers, there was a trend toward a significant increase in IS risk as the number of elevated biomarkers increased from 0 to 2.

However, the current research on the dysregulation of epigenetic mechanisms by Hcy, which mediates the development of IS, is mainly focused on DNA methylation, and there are fewer related studies. Thus, it is worthwhile to further explore the research.

## 4 Pathologic Basis of HHcy‐Related IS

Various factors, such as cofactor nutritional disorders and/or mutations in key enzyme genes, result in impaired Hcy metabolism, which further leads to elevated levels of Hcy in the blood, triggering HHcy. High levels of Hcy affect the onset and development of IS through mechanisms related to inflammation and oxidative stress, neurotoxicity, and/or dysregulation of epigenetic mechanisms. High levels of Hcy, through all these mechanisms, can trigger various pathologic processes such as vascular injury, thrombosis, and/or decreased blood flow, which can lead to IS and aggravate its poor prognosis (Kaul et al. 2006).

### 4.1 Vascular Damage

**FIGURE 3:**
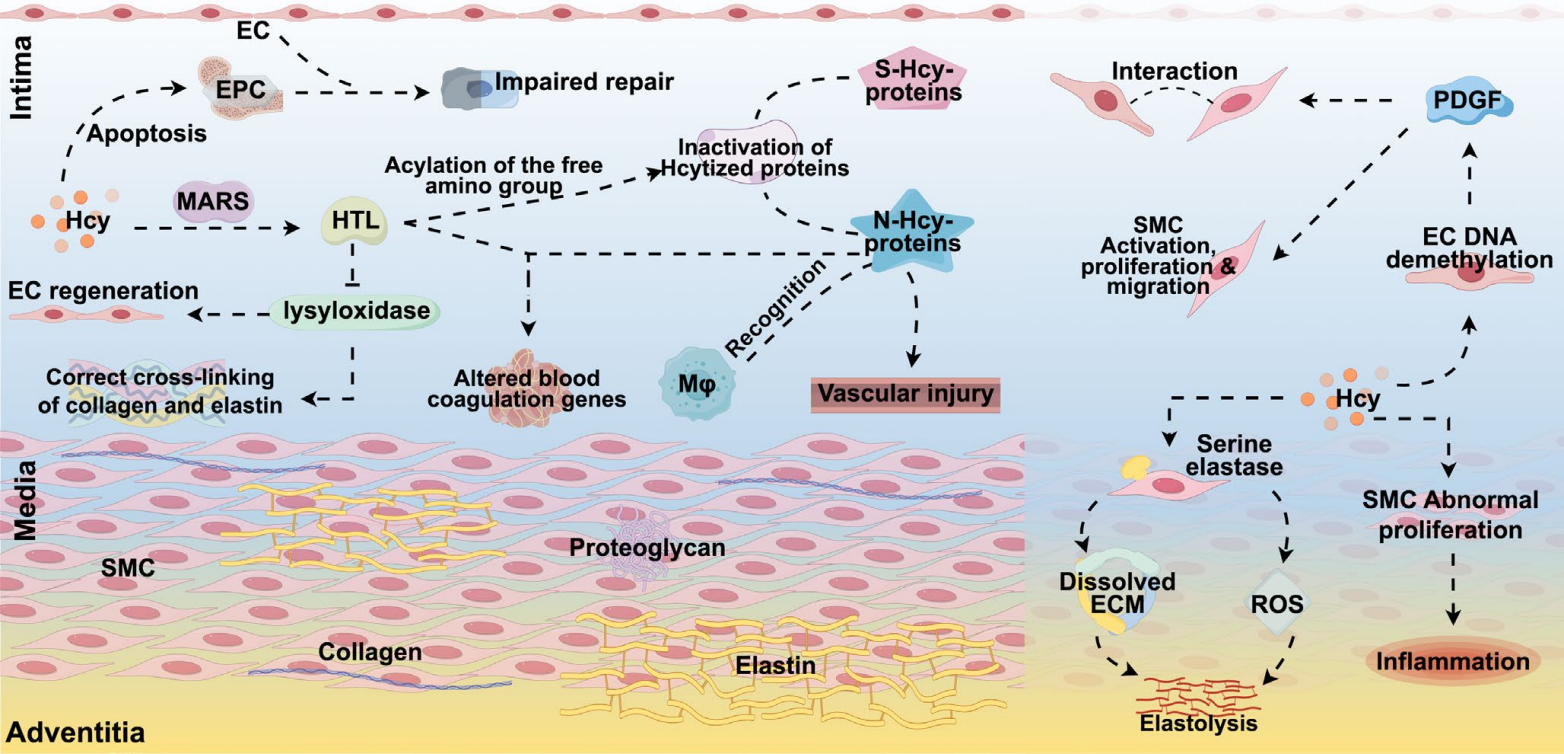
Vascular damage. Homocysteine causes endothelial cell apoptosis, protein modification, abnormal proliferation of smooth muscle cells, etc.; at the same time, platelet‐derived growth factor, elastase, etc. promote smooth muscle cell migration, inflammation, and elastase hydrolysis; these processes jointly lead to vascular structure damage and functional impairment.

It is well known that the arterial vasculature is divided into three layers, namely: (i) endothelial, (ii) mesothelial, and (iii) ectothelial. (i) The intima is the innermost layer of the blood vessel, the thinnest of the three layers, and consists of EC, which plays a crucial role in maintaining vessel wall homeostasis by regulating vascular tone, permeability, inflammation, and cell growth (Mallika et al. 2007). Thus, impaired EC can have a major adverse effect on vascular stability and function, and this process of perturbing EC is known as endothelial dysfunction and underlies the development of a series of related events regarding AS lesions (Esse et al. 2019). (ii) The middle layer of the vasculature, located between the intima and the peritoneum, consists mainly of interconnected smooth muscle cells (SMCs) separated by continuous elastin filaments, collagen, and proteoglycans, which are important for maintaining vascular diastolicity (Ramos et al. 1998). To a large extent, the middle layer is responsible for the structural and elastic properties of the arteries; therefore, processes causing damage to any component of the middle layer leading to remodeling of the middle layer may affect the structural and functional integrity of the arteries leading to deleterious vascular disease processes (Tsai et al. 1994). (iii) The outer membrane of blood vessels consists mainly of loose connective tissue and elastic and collagen fibers (Huehns et al. 1996; Figure 3).

Hcy can damage the vasculature through oxidative stress, inflammatory responses, and other related mechanisms, leading to vascular dysfunction, which contributes to the development of AS as well as a range of other IS‐related pathological processes (Liu et al. 2019). A study by Eikelboom et al. (2000) showed that elevated plasma Hcy had a close‐graded relationship with IS due to atherosclerosis of the large arteries, to a lesser extent with the disease of the small arteries and was not associated with myocardial embolism or other etiologic causes of IS subtypes. This suggests that the deleterious effects of Hcy are mediated primarily through the promotion of AS. Dai et al. (2019) evaluated the relationship between Hcy and AS in IS‐associated vascular beds (e.g., intracranial arteries, extracranial carotid arteries, and the aortic arch) using three‐dimensional magnetic resonance vessel wall imaging. Hcy was found to be independently associated with IS‐related vascular AS plaques, suggesting that Hcy may be an independent risk factor for vascular AS. Feng et al. (2023) investigated the effect of lipoprotein(a) [Lp(a)] on stroke recurrence at 1 year in patients with IS. The study showed that elevated Lp(a) levels were independently associated with stroke recurrence within 1 year in patients with IS; there was an interaction between Lp(a) and Hcy in stroke occurrence, especially in the large atherosclerotic (LAA) subtype. This suggests that in our secondary prevention, we should be aware that elevated Hcy may exacerbate Lp(a)‐related stroke recurrence. A study by Shi et al. (2018) found that the risk of IS recurrence was 1.76 times higher in patients in the high Hcy group than in those in the low Hcy group. Subgroup analysis showed a significant correlation between high Hcy levels and IS recurrence in patients with atherosclerotic ischemia of the large arteries. The above two studies suggest that Hcy is a risk factor for the risk of relapse in patients with large atherosclerotic IS.

It has been shown that elevated Hcy levels increase vascular oxidative stress and promote inflammation in the arterial wall, which triggers a series of histopathologic responses to vascular injury, including EC injury and SMC activation, all of which events are involved in the development and progression of AS (Upchurch et al. 1997). (i) EC impairment: a key event in the vascular pathology associated with elevated plasma Hcy levels is the impairment of normal EC function, probably because Hcy interferes with cellular redox signaling, thereby increasing the incidence of AS through EC function impairment and participating in the pathogenesis of IS (Weiss 2005). EC dysfunction is the first step in the development of AS lesions, which is subsequently accompanied by vascular inflammation, which leads to the formation of AS plaques (Ross 1999). One of the mechanisms by which Hcy affects the vascular endothelium is through irreversible protein homocysteinylation. Through the mis‐editing function of methionyl‐tRNA synthetase (MARS), Hcy can be converted to HTL, a chemically active metabolite that acylates free amino groups, a reaction that generates Hcy‐ized proteins that do not retain their biological activity (H. Jakubowski 1997). In addition to this, Alam et al. (2009) showed that Hcy decreases the number of endothelial progenitor cells (EPC) in IS patients through apoptosis. Ischemic injury activates the migration of EPC from the bone marrow to repair damaged sites by direct incorporation of EPC or repopulation with mature EC, but elevated levels of Hcy lead to apoptosis of EPC, which reduces the number of EPC. (ii) SMC activation: vascular SMC (VSMC) proliferation is directly stimulated by Hcy and is a key component of AS (Pines et al. 2002). HHcy induces the development of AS by inducing abnormal proliferation of SMC and increasing the inflammatory process (Selhub and D'angelo 1998). HHcy stimulates VSMC proliferation and also induces the formation of serine elastase in VSMC, which leads to elastolysis by lysing the extracellular matrix and generating ROS, all of which events contribute to the onset and progression of AS lesions (Rabelo et al. 2022). (iii) EC and SMC interaction: HHcy is an independent risk factor for AS as it promotes EC dysfunction and VSMC activation (Harker et al. 1976). However, little is known about the interactions between EC and VSMC under HHcy conditions. Zhang et al. (2012) investigated whether Hcy activates VSMC in humans and mice by increasing EC secretion of mitogenic platelet‐derived growth factor (PDGF). In this study, the authors found that an increase in Hcy levels did not affect VSMC activity for 24 h until Hcy concentrations reached 500 μM; in contrast, 100 μM Hcy significantly promoted the proliferation and migration of VSMC co‐cultured with human EC, and pretreatment with a PDGF receptor inhibitor partially reversed this effect. The upregulation of PDGF by Hcy was similarly confirmed in the aortic intima of HHcy mice. This is because Hcy decreased the expression and activity of DNA methyltransferase 1 (DNMT1), increased the demethylation of the PDGF‐A, ‐C, and ‐D promoters, and enhanced the promoter‐binding activity of the transcription factor SP‐1 so that Hcy could concentration‐dependently upregulate the mRNA levels of PDGF‐A, ‐C, and ‐D in EC. This experiment demonstrated that Hcy could upregulate PDGF levels and paracrine secretion through DNA demethylation of EC, affect the cross‐talk between EC and VSMC, and lead to the activation of VSMC, increasing the proliferation and migration of VSMC.

It is well known that the blood vessels that make up the central nervous system (CNS) vascular system have a unique feature called the BBB, which precisely controls CNS homeostasis through the barrier effect of the BBB, enabling neurons to perform appropriate functions and protecting neural tissue from toxins and pathogens (Keaney and Campbell 2015). Chronic elevation of plasma Hcy alters vascular mature EC function and activates thiolation and homocysteinylation of plasma proteins and enzymes through important pathological and biochemical modifications, thereby deleteriously affecting cerebral vascular permeability and, ultimately, brain parenchyma. The reason why HHcy can be a direct cause of brain tissue damage is that, on the one hand, Hcy alters the structure and function of mature EC in the BBB and increases their permeability; on the other hand, Hcy alters the function of astrocytes by affecting them, and astrocyte and mature EC alterations promote each other, accelerating the damage of HHcy to brain tissue (Beard et al. 2011). One study demonstrated that a high Hcy diet can lead to capillary damage in the CA1 region of the hippocampus, as evidenced by localized irregular thickening of the basement membrane, mitochondrial and cytoplasmic swelling, and the appearance of fibrosis in the CA1 region of the hippocampus (Lee et al. 2005). Another study showed that elevated Hcy levels produce significant toxic effects on cerebral microvessels in the brain (Kamath et al. 2006). Based on these findings, it seems reasonable to consider a link between HHCy and BBB integrity.

Hcy can induce BBB disruption, and this disruption may be caused by Hcy leading to an imbalance in the activities of matrix metalloproteinase 9 (MMP‐9) and tissue inhibitor of metalloproteinases 4 (TIMP‐4), that is, an increase in the activity of MMP‐9 and a decrease in the activity of TIMP‐4, which subsequently interacts with the different components of the BBB leading to the disruption of this structure (Tyagi et al. 2009). Kalani et al. (2014) showed that miRNA29b regulates DNA methyltransferase 3b (DNMT 3b) activity, which ultimately leads to MMP‐9 activation to digest extracellular matrix and connexin, resulting in vascular leakage.

### 4.2 Thrombosis

**FIGURE 4:**
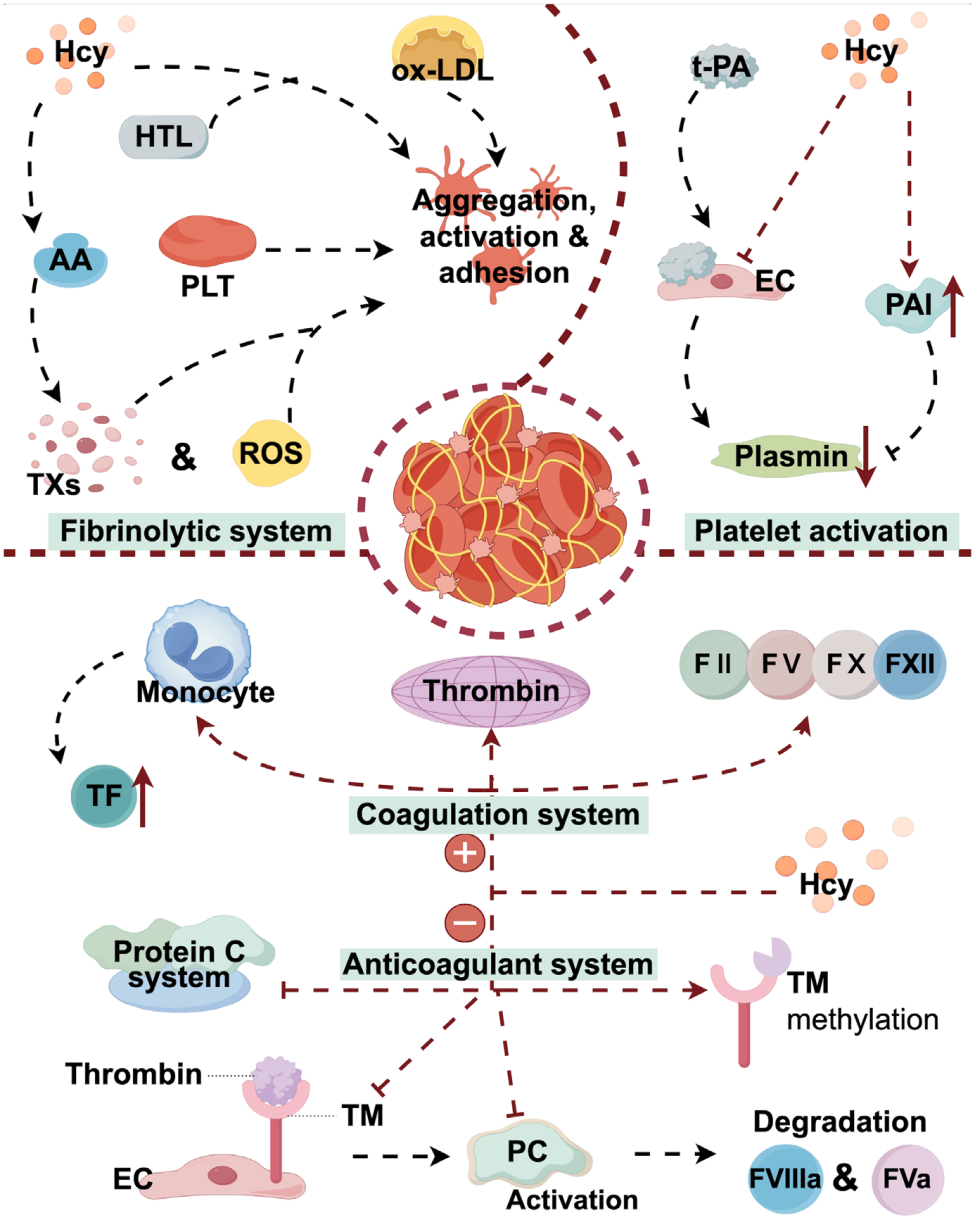
Thrombosis. Homocysteine can activate platelets, enhance the activity of coagulation factors, and inhibit plasmin activity, thereby promoting thrombosis. It also damages endothelial cells through oxidative stress, intensifying the coagulation process.

HHcy may be involved in the pathogenesis of IS by increasing blood coagulability and promoting thrombosis through mechanisms such as EC injury and coagulation abnormalities (Coppola et al. 2000). Chen et al. (2023) saw a male patient in his 40s with IS in the left middle cerebral artery supply region who also had a combination of multifocal extracranial venous and arterial thrombosis. He had a significant past medical history of Crohn's disease and anabolic steroid use, a negative young stroke screen in addition to severely elevated Hcy concentrations, folate, and VB12 deficiencies, and further testing revealed that he was an MTHFR C677T variant. This case suggests that the etiology of massive IS and combined venous and arterial thrombosis may be due to a hypercoagulable state caused by significantly elevated Hcy levels. Sikora et al. (2024) analyzed plasma and urine from 191 stroke patients and 291 healthy individuals to study the relationship of sulfur‐containing amino acid metabolites with fibrin clot dissolution time and stroke. Sulfur‐containing amino acid metabolites such as urinary homocysteine‐thiolactone, urinary glutathione, and plasma cysteinylglycine were found to be associated with both fibrin clot properties and stroke, suggesting that these metabolites may induce stroke by promoting unfavorable fibrin clot properties. Other sulfur‐containing amino acid metabolites such as urinary homocysteine, urinary cysteine, plasma cysteine, and the MTHFR C677T polymorphism were associated with stroke but did not affect fibrin clot properties. This study suggests that some of the sulfur‐containing amino acid metabolites may be pro‐thrombotic and increase the incidence of stroke. Li, Wang, et al. (2020) analyzed 3044 patients from 73 pre‐specified clinical sites based on the CHANCE trial (Clopidogrel for the Treatment of Patients at High Risk of Acute Non‐Disabling Cerebrovascular Events). The study found a significant interaction between Hcy levels and randomized antiplatelet therapy for recurrent stroke in women but not in men after adjusting for confounders. The reasons for the results of this experiment may lie in the fact that (a) estrogen regulates Hcy levels, leading to a different distribution of Hcy levels between females and males, which in turn leads to differences in randomized antiplatelet therapy between genders, and (b) patients with elevated Hcy levels are less sensitive to antiplatelet therapy (Figure 4).

Together, the above clinical studies suggest that Hcy may contribute to thrombosis through multiple mechanisms, which in turn influences the onset and progression of IS. However, the molecular mechanisms underlying the pro‐thrombotic effects of Hcy are not fully understood and may involve oxidative stress, DNA hypomethylation, and pro‐inflammatory effects (Undas et al. 2005). However, patients with HHcy are pathologically characterized by (i) vascular damage and platelet activation leading to thrombosis, (ii) an imbalance in the coagulation and anticoagulation systems, and impairment of the fibrinolytic system leading to thrombosis and impaired thrombolysis (Bienvenu et al. 1993). Hcy leads to EC stripping, exposing the subEC matrix to the luminal surface, leading to platelet activation and thrombus formation, which is manifested as platelet aggregation and formation of platelet‐rich thrombi at the site of EC injury in animal models and humans (Harker et al. 1974). In addition, it has also been shown that Hcy also promotes the expression of coagulation factors II, V, X, and XII and decreases the activation of protein C and antithrombin III in cell cultures (Harpel et al. 1996), as well as possibly the impaired fibrinolytic disorder and impaired fibrinolytic capacity that accompanies HHcy (Sauls et al. 2006).

(i) Vascular injury and platelet activation: (a) Vascular injury: under in vivo conditions, Hcy‐mediated EC injury is most commonly characterized by EC dysfunction, manifested by impaired EC‐dependent vasodilatation, along with the development of a pro‐thrombotic and pro‐inflammatory phenotype in the EC (Austin et al. 2004). In addition, a recent report extensively examined the effects of different Hcy metabolites on HUVEC and showed that both HTL and protein N‐homocysteinylation have the potential to perturb vascular homeostasis by altering the expression of genes involved in coagulation (Gurda et al. 2015). HTL, a highly active derivative of Hcy, may also be associated with vascular injury and subsequent thrombosis (H. Jakubowski 2004). It has been shown that HTL reacts with proteins by forming amide bonds with the amino groups of lysine residues, which leads to the generation of Nε‐homocysteinylated proteins with altered structural and biochemical properties (Glowacki and Jakubowski 2004). Undas et al. (2004) provided evidence that antibodies to Nε‐homocysteinylated albumin and hemoglobin are present not only in healthy individuals but also in stroke patients, a finding that suggests that the formation of such antibodies against self‐antigens in patients with HHcy may be involved in endothelial cell damage by Hcy. (b) Platelet activation: in the later stages of the AS process, Hcy increases platelet activation and aggregation and leads to coagulation abnormalities, triggering thrombosis, which leads to vascular occlusion and promotes IS (Romecín et al. 2017). Increased platelet activation has been reported to be associated with increased thromboxane production in patients with homocystinuria (Di Minno et al. 1993). Elevated Hcy levels enhance arachidonic acid‐like metabolism and promote overproduction of thromboxane, leading to increased platelet adhesion and inadequate platelet inhibition (Signorello et al. 2002). Signorello et al. (2002) showed that Hcy induces the release of arachidonic acid and that incubation of platelets with Hcy significantly increases basal levels of thromboxane B2 (TXB2) and ROS, an effect that is time‐ and dose‐dependent. An imbalance in the platelet redox state and an increase in TXB2 formation may produce excessive activation of platelets, leading to a state of thrombosis that can trigger IS. Davì et al. (2001) demonstrated that the formation of 8‐isoprostane F2α, a platelet‐activating product of arachidonic acid peroxidation, was significantly correlated with TXB2 levels, and hypothesized that enhanced peroxidation of arachidonic acid might be an important mechanism of platelet activation in homocystinuria purebred subjects with markedly elevated Hcy levels. Other mechanisms include Hcy, which enhances platelet response to thrombin in a dose‐dependent manner, a phenomenon associated with modulation of L‐arginine transport and reduced NO formation (Leoncini et al. 2003). It has also been shown that Hcy and oxidized LDL enhance platelet adhesion to EC in the blood flow state (Dardik et al. 2000). In addition to this, the experimental results of Malinowska et al. (2012) showed that Hcy and HTL stimulate the adhesion of activated platelets to collagen and fibrinogen. In addition, exposure of platelets to HTL resulted in a stronger modulation of platelet adhesion than treatment of platelets with Hcy. This suggests to us that Hcy and HTL may influence platelet adhesion.

(ii) Imbalance of the coagulation and anticoagulation systems, disorders of the fibrinolytic system: (a) activation of the coagulation system: A study by Undas et al. (2005) showed that Hcy stimulates thrombosis by promoting thrombin formation through the exogenous coagulation activation pathway, thereby increasing the risk of initial and recurrent stroke. Khajuria and Houston (2000) demonstrated Hcy‐induced monocyte tissue factor (TF) expression under different methodological conditions, and according to them, it was hypothesized that EC and AS plaque‐derived monocytes or macrophages could be the source of detectable TF in plasma obtained from AS subjects. (b) Inhibition of the anticoagulant system: the protein C system, which includes protein C, protein S, and thrombomodulin (TM), is considered to be the most important system for anticoagulation in the microvascular system (Weiler and Isermann 2003). Hcy has been shown to inhibit protein C activation by direct reduction of disulfide bonds in the structural domain of epidermal growth factor in the protein C molecule (Lentz and Sadler 1991). In bovine arterial and venous cells, Hcy at concentrations of 0.2–10 mM has been found to reduce TM activity by approximately 50% compared to baseline values (Rodgers and Conn 1990). In contrast, TM is a thrombin receptor on the EC surface that forms a complex with thrombin, leading to activation of protein C. Activated protein C is an important endogenous anticoagulant that downregulates coagulation by breaking down coagulation factors Va and VIIIa (Sadler 1997). Therefore, TM may have a protective effect on IS by decreasing coagulation. Yang et al. (2016) found that TM methylation levels were higher in IS patients than in controls, which was positively correlated with plasma Hcy levels and negatively correlated with mRNA expression of TM. This study suggests that HHcy leads to hypermethylation of the TM gene and further induces silencing of the TM gene, which may play an important role in the development and progression of HHcy‐associated IS since TM can play a protective role against IS by down‐regulating coagulation. (c) Fibrinolytic system disorders: Hcy can affect the fibrinolytic cascade (Pines et al. 2002). Hcy affects fibrinolytic activity by inhibiting the binding of EC and tissue‐type fibrinogen activators (Mccully 2015).

In conclusion, HHcy renders human EC and VSMC more susceptible to injury, which leads to increased TF expression, coagulation factor activation, increased thrombin generation, and expression of fibrinogen activator inhibitors; this, in turn, inhibits heparin synthesis, TM expression, and synthesis of tissue‐type fibrinogen activator, and the combined effect leads to platelet aggregation/thrombosis, and subsequently, IS (Hainsworth et al. 2016).

Clinical and experimental evidence suggests that circulating Hcy is pathophysiologically linked to atrial fibrillation (AF) and may be a marker of this arrhythmia (Marcucci et al. 2004). Hcy may contribute to the development of AF through multiple mechanisms, including electrical remodeling and structural remodeling (Ay et al. 2003). (i) Electrical remodeling: Hcy may directly affect potassium currents in atrial myocytes because Hcy affects K^+^ channels, which inhibit Ito (Ito channels may have functional residues that are regulated by Hcy and/or cysteine, which directly lead to channel inhibition) and IKur currents while increasing IK1 currents (Zhang et al. 2005). In addition, elevated Hcy levels cause a significant increase in the inward current (sodium current) by slowing down the repolarization process (decreasing the repolarizing potassium current) and facilitating the depolarization process (increasing the depolarizing sodium current) in human atrial myocytes, which leads to membrane depolarization during phases 2 or 3 of the action potential, prolongs the action potential duration, and leads to an early post‐depolarization of the resting membrane potential of the myocyte, thus triggering focal ectopic electrical activity in cardiomyocytes (Cai et al. 2009). (ii) Structural remodeling: In addition to electrical remodeling, Hcy causes structural remodeling of the atria (Perła‐Kaján et al. 2007). Atrial structural remodeling is a complex process involving activation of MMP‐9 and extracellular signal‐regulated kinases: HHcy leads to extracellular matrix remodeling through activation of the extracellular signal‐regulated kinase/MMP‐9 signaling axis (Moshal et al. 2006); furthermore, among extracellular matrix proteins, type I collagen synthesis is altered by HHcy, and subsequent atrial fibrosis leads to slow and heterogeneous atrial conduction, favoring the emergence of a fragile reentrant matrix (Shimano et al. 2008). In addition to this, HHcy increases blood coagulability, favoring atrial thrombosis, which in turn leads to IS (Yao et al. 2018).

The studies described above suggest that elevated Hcy levels appear to be involved in structural or electrophysiological remodeling of the heart, as it is associated with enlarged left atria, increased myocyte size, cardiac fibrosis, and remodeling of sodium/potassium ion channels, leading to the development of a cardiac environment that is susceptible to AF; combined with the fact that Hcy also activates the pro‐thrombotic tendency of blood in the atria, the two act together to increase the chances of developing IS.

### 4.3 Decreased Blood Flow

**FIGURE 5:**
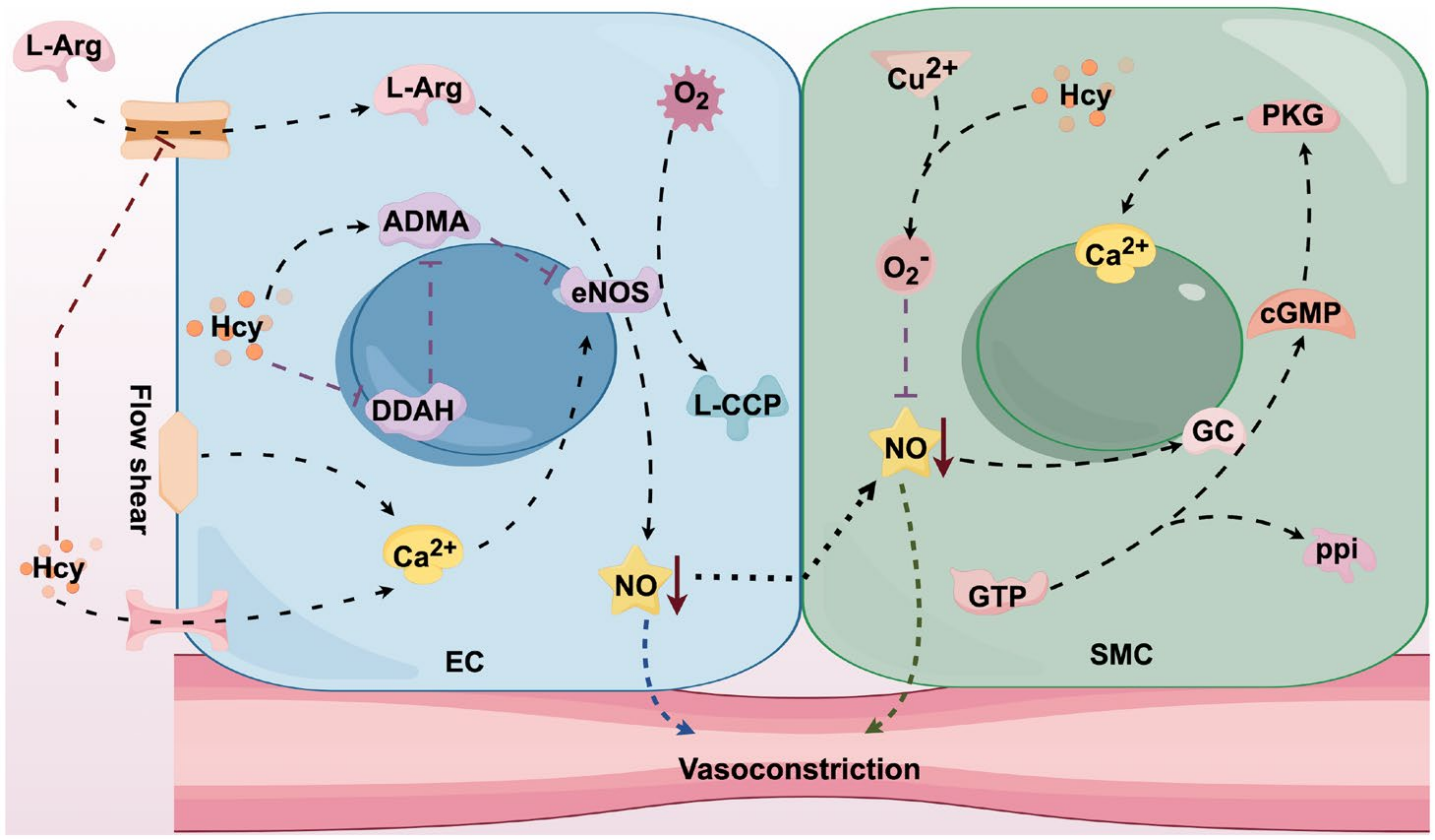
Vasoconstriction. Homocysteine interferes with the uptake of L‐arginine by endothelial cells, inhibits the activity of endothelial nitric oxide synthase, promotes oxidative stress, reduces the generation of nitric oxide, causes smooth muscle cell contraction, and thereby triggers vascular contraction.

In addition to the fact mentioned above that Hcy can induce intravascular thrombosis, which in turn leads to cerebral vascular occlusion and reduced cerebral blood flow, triggering IS, Hcy also has toxic effects on cerebral blood vessels, which may lead to cerebral vasoconstriction and reduced cerebral blood flow, thus increasing the risk of IS development (Williams et al. 2014; Figure 5).

The vascular endothelium consists of the EC, and various stimuli act on the EC to produce vasoactive substances, which include vasodilating factors (prostacyclin, endothelium‐derived hyperpolarizing factor, etc.) as well as vasoconstricting factors (thromboxane, endothelin, etc.), which can affect vasodilatory and constrictive functions, thereby regulating blood flow to the tissues (Yanagisawa et al. 1988). It has been shown that the presence of EC in isolated arterial preparations is essential for acetylcholine‐induced vasodilation, an effect attributed to substances released by EC, namely nitric oxide (NO) (Ignarro et al. 1987).

NO is produced by endothelial‐type nitric oxide synthase (eNOS) catalyzing its precursor L‐arginine. Under physiological conditions, NO production diffuses across the vascular endothelium into vascular smooth muscle cells, activating guanylate cyclase, which produces cyclic guanosine monophosphate and mediates vasodilatory effects. Another important stimulus is the shear stress generated by flowing blood, which can lead to the activation of ion channels for a rapid response or induce a sustained release of NO through a phosphorylation process to maintain vasodilation (Vallance and Chan 2001).

In previous studies, elevated Hcy levels have been strongly associated with EC dysfunction due to oxidative stress or subclinical inflammation, and if EC function is impaired, the BBB is impaired, and the cerebral vasculature loses its ability to autoregulate vasodilatation, which can lead to a more severe initial IS and disruption of recovery (Nam et al. 2019). It has been shown that Hcy causes direct damage to the EC both in vitro and in vivo, and clinically, this manifests itself as impaired blood flow‐mediated vasodilatation, mainly due to reduced synthesis and bioavailability of the vasodilator factor NO, which interferes with cerebral blood flow (Li et al. 2002).

Asymmetric dimethylarginine (ADMA) is an inhibitor of eNOS (Sydow et al. 2003), and dimethylarginine dimethylamine hydrolase (DDAH) is a catabolic enzyme of ADMA (Dayal et al. 2008). HHcy is a pathogenetic mechanism that leads to circulatory disturbances in the brain and other blood vessels because Hcy activates ADMA on the one hand and degrades DDAH on the other hand, both of which work together to reduce eNOS activity, leading to a decrease in NO concentration, which impairs vasodilatory capacity (Do Carmo et al. 2016). Toda and Okamura (2016) suggested that Hcy increases ADMA production and impairs NO synthesis in the EC, which diminishes the vasodilatory effect of NO, and this leads to impaired cerebral circulation, which is an important predisposing factor for IS; diminished nitrogen oxides and insufficient cerebral perfusion lead to increased amyloid‐β production, which inhibits EC function, thus creating a vicious circle.

In addition to this, Hcy, like other sulfhydryl amino acids, reacts readily with NO to form S‐nitroso‐Hcy, and in this way, Hcy inactivates NO (D'emilia and Lipton 1999). Early studies of the cerebral microcirculation observed that in anesthetized rats, hyperinflation of Hcy‐Cu^2+^ resulted in a superoxide‐mediated reduction in resting cerebral blood flow as well as EC‐dependent and NO‐mediated attenuation of the response, whereas Cu^2+^ alone did not, and that concomitant perfusion of superoxide dismutase (SOD) prevented the effects of Hcy‐Cu^2+^ (Zhang et al. 1998). This suggests that superoxide O_2_^−^ produced by the reaction of Hcy with the metal Cu^2+^ inhibits NO‐related cerebrovascular responses by scavenging NO and reducing the dose of NO used for vasodilation. However, because NO is beneficial to the cerebral vasculature and antiplatelet effects in the early stages of cerebral ischemia, Hcy‐induced NO scavenging may be one of the mechanisms by which Hcy worsens the outcome of IS.

Lai and Kan (2015) summarized the potential cellular mechanisms by which Hcy leads to impaired EC function, particularly the effect of HHcy on NO, suggesting that multiple pathways may be involved: Hcy‐induced impaired transport of the NO precursor L‐arginine into endothelial cells upregulates ROS production by NADPH oxidase, potentially derailing the NO‐generating reaction and generating more ROS in a process known as eNOS uncoupling; a vicious cycle of further uncoupling and eNOS destruction leads to decreased NO bioavailability and inflammation; Hcy also degrades DDAH, leading to the accumulation of the eNOS inhibitor ADMA; in a separate reaction, Hcy generates HTL, which attacks lysine‐rich proteins and may trigger an apoptotic response associated with EC stress.

These mechanisms described above suggest to us that the negative effects of Hcy on EC function and NO bioavailability may help to explain the Hcy‐induced vasodilatory dysfunction triggered by IS.

Hcy‐induced EC dysfunction is the basis of all pathological processes, and the resulting processes of AS, BBB destruction, thrombosis, and vasoconstriction can ultimately lead to the development of IS and aggravate the poor prognosis of IS.

## 5 Prevention and Treatment

Studies have shown that folate is beneficial in various neurological disorders because it promotes cell signaling and neurogenesis and reduces Hcy levels in the blood (Davis et al. 2018). Davis and Rajanikant (2020) showed that folate supplementation after cerebral ischemia promotes neuronal survival and neural regeneration and regulates ischemic injury‐induced cell death, acidic organelle formation, and mitochondrial membrane depolarization in vitro. Folate supplementation after ischemia also promotes cell proliferation and axonal growth. These results suggest that folate can potentially repair and rescue nerve cells from ischemia/reperfusion injury.

However, most mammals, including humans, are unable to synthesize folate and must, therefore, obtain it from dietary sources such as green vegetables, certain citrus fruits, liver, and whole grains (Serrano‐Amatriain et al. 2016). The major dietary folate is 5‐MTHF; VB_12_‐dependent MS plays an important role in facilitating the conversion of extracellular 5‐MTHF to tetrahydrofolate; in addition, VB_6_ is required for the formation of 5,10‐methylenetetrahydrofolate from tetrahydrofolate (Yang et al. 2024). Cells throughout the body, including neurons and glial cells, express folate transporters, suggesting that folate and vitamin B12, as well as VB6, play a crucial role in Met and Hcy metabolism in most cell types (Sirotnak and Tolner 1999).

Nutritional deficiencies in the vitamin cofactors required for Hcy metabolism (folate, VB_12_, and VB_6_) can lead to HHcy, and significantly elevated Hcy concentrations have been observed in patients deficient in the essential cofactors VB_12_ and folate (Brattström et al. 1988). Zhang and Zhang (2023) found that plasma Hcy levels were positively associated with stroke risk, especially in the context of LAA and small artery occlusion (SAO) strokes. These findings suggest that therapies using vitamin cofactor supplementation to lower Hcy are of potential clinical relevance in the prevention of stroke, especially IS (LAA, SAO subtypes).

In IS patients with mild or mildly elevated Hcy levels, lowering Hcy with high‐dose vitamin therapy did not reduce the risk of IS recurrence (Toole et al. 2004). Therefore, further studies are necessary to verify whether the risk of IS in patients can be reduced by intensive control or by maintaining lower Hcy levels through folate and VB_12_ supplementation. The Mendelian randomization study by Yuan et al. (2021) provided evidence that lowering Hcy levels is generally beneficial in the prevention of various cardiovascular diseases in the general population. The findings confirm and extend the evidence that Hcy‐lowering B‐vitamin therapy may play a role in the prevention of stroke, particularly IS. In several clinical trials, the dose of folate used to reduce Hcy varied from doses below the recommended daily allowance (RDA, 0.4 mg/day) to very high doses, such as 5 mg/day or even higher (Shirodaria et al. 2007). It has been shown that oral folate (0.5–5.0 mg/day) reduced fasting Hcy levels by 25%–30%, whereas supplementation with VB_12_ (0.02–1 mg/day) reduced Hcy levels by a further 7%, and VB_6_ had no effect on fasting Hcy levels, but significantly reduced HHcy after Met loading (Moens et al. 2008).

However, it is worth noting that large randomized studies such as the VISP trial (Toole et al. 2004), the HOPE‐2 trial (Saposnik et al. 2009), and the VITATOPS trial (Vitatops Trial Study Group 2010) examined the effects of long‐term administration of folate, VB6, and VB12 on cardiovascular risk, and preliminary analyses suggest that vitamin therapy has failed to achieve a significant preventive effect (Table 1).

**TABLE 1:** Clinical trials of a vitamin intervention to reduce the risk of ischemic stroke in patients with hyperhomocysteinemia.

Clinical trial	Vitamin cofactor (mg/day)			Follow‐up period (Year)	Number of patients	Conclusion
	Folate	VB_12_	VB_6_			
VISP (Toole et al. 2004)	2.5	0.4	25	7	3680	Ineffective
HOPE‐2 (Saposnik et al. 2009)	2.5	1	50	5	5522	Effective
VITATOPS (Vitatops Trial Study Group 2010)	2	0.5	25	9	8164	Ineffective

Abbreviations: HOPE‐2, heart outcome prevention evaluation‐2; VISP, vitamin intervention for stroke prevention; VITATOPS, vitamins to prevent stroke.

The aim of the VISP trial (Toole et al. 2004; Vitamin Intervention for Stroke Prevention) was to compare the effect of high‐dose versus low‐dose Hcy‐lowering therapy on the risk of recurrent stroke and death. The trial, which began in September 1996 and ended in May 2003, involved 3680 adults with non‐disabling IS at 56 university hospitals, community hospitals, private neurology clinics, and Veterans Affairs medical centers in the United States, Canada, and Scotland, who were randomly assigned to receive either high‐dose (25 mg VB6, 0.4 mg VB12, and 2.5 mg folate) or low‐dose (200 μg VB6, 6 μg B12, and 20 μg folate) Hcy‐lowering treatment once daily. The study found that during a 2‐year follow‐up period, the mean Hcy reduction in the high‐dose group was two micromol/L higher than in the low‐dose group, but high‐dose Hcy‐lowering therapy did not produce a significant effect on the pre‐specified endpoints (primary endpoint: recurrent cerebral infarction; secondary endpoint: death). However, the study did not include a placebo‐treated group; therefore, the study raises the hypothesis that low‐dose treatment may produce the greatest benefit, while further increases in dose produce no additional effect.

The aim of the HOPE‐2 trial (Saposnik et al. 2009; Heart Outcome Prevention Evaluation‐2) was to determine whether vitamin therapy reduces the risk of ischemic and hemorrhagic stroke, as well as stroke‐related disability. Researchers followed 5522 patients with vascular disease or diabetes for 5 years and evaluated the effect of Hcy‐lowering therapy with folate (2.5 mg/day), VB6 (50 mg/day), and VB12 (1 mg/day) on cardiovascular risk. Although the study showed that vitamin therapy reduced the risk of nonfatal stroke, it failed to demonstrate any effect of Hcy‐lowering therapy on neurologic deficits at 24 h or functional dependence at discharge and 7 days. Thus, although this trial showed that Hcy lowering by folate, VB6, and VB12 did reduce overall stroke risk, it did not reduce stroke severity or disability and failed to address the question of whether low‐dose Hcy‐lowering therapy affects cardiovascular risk.

The aim of the VITATOPS trial (Vitatops Trial Study Group 2010; VITAmins TO Prevent Stroke) was to assess whether once‐daily supplementation with B vitamins in addition to usual medical care would reduce total Hcy and decrease the combined incidence of nonfatal stroke, nonfatal myocardial infarction, and vascular death in patients with recent stroke or transient ischemic attack. Trial researchers collected 8164 patients with a recent (within the past 7 months) stroke or transient ischemic attack from 123 medical centers in 20 countries who were randomly assigned to receive either B vitamins (4089) or a placebo (4075) between November 19, 1998 and December 31, 2008, with patients in the B vitamin‐treated group took B vitamins (2 mg folate, 25 mg VB6, and 0.5 mg VB12) daily. The findings suggest that daily folate, VB6, and vitamin B12 are safe in patients with recent stroke or transient ischemic attack but do not appear to be more effective than placebo in reducing the incidence of major vascular events. The trial results do not support the use of B vitamins to prevent stroke recurrence.

One of the reasons why folate supplementation may not be as effective as expected is that there is a “feedback” mechanism in the methionine cycle (Lieber and Packer 2002): SAHH is a bi‐directional enzyme that, under normal conditions, favors hydrolysis of SAH to Hcy; however, in the presence of increased Hcy, this enzyme activity is reversed, resulting in accumulation of SAH. In addition, SAH, as a demethylation product of SAM transmethylation, is itself a potent competitive inhibitor of SAM‐mediated methylation reactions, and thus, the decrease in intracellular methylation reactions may be the result of either a decrease in SAM formation or an increase in SAH (Xiao et al. 2015). In turn, alterations in cellular methylation potential have an impact on gene expression, with the expression of several AS‐causing molecules being up‐regulated through hypermethylation of the promoter regions of several AS‐causing genes; furthermore, folate contributes to the deterioration of AS by promoting cell proliferation through its role in thymidine synthesis (Loscalzo 2006). There is also the fact that increased methylation of arginine residues raises levels of ADMA, which inhibits or uncouples eNOS, thereby adversely affecting clinical outcomes (Mittermayer et al. 2006).

It has also been suggested that the ineffectiveness of vitamin therapy in preventing stroke is due to the accumulation of cyanocobalamin byproducts, which is more pronounced in patients with renal insufficiency because of their poor metabolite clearance (Spence et al. 2017). Liu et al. (2023) extracted data from IS patients admitted to the Chinese Stroke Center Consortium from 2015 to 2019 and analyzed patient characteristics and in‐hospital mortality to investigate the relationship between blood Hcy and in‐hospital mortality in HHcy patients and normal patients. HHcy was found to correlate with in‐hospital mortality in IS patients, but this correlation disappeared after controlling eGFR, suggesting that HHcy is a marker of renal dysfunction. This study suggests that prevention and management of renal damage may be an important measure to reduce mortality in patients with HHcy after IS. Using data from 11 large centers in Korea, Nam et al. (2022) found that high plasma Hcy levels were associated with poor prognosis in patients with AF‐related stroke and that this relationship showed different patterns depending on the presence of renal dysfunction. In patients with AF‐related stroke, renal function not only affects plasma Hcy levels but also appears to influence the relationship between Hcy and IS prognosis. This study suggests that the role of vitamin therapy in the prevention of IS should be reassessed and will help to categorize the appropriate high‐risk population for vitamin therapy. Brown et al. (2023) discussed the effects of high‐dose folate and cyanocobalamin toxicity in patients with renal failure and the MTHFR C677T subgroup. Studies have shown that the toxicity of folate and cyanocobalamin is due to the lack of bioequivalence with the natural dietary forms (L‐methyl folate and methylcobalamin) and that low doses of folate and cyanocobalamin are safer than high doses of folate and cyanocobalamin in these subgroups of patients, whereas L‐methyl folate and methylcobalamin, which have a lower level of toxicity, are more effective in lowering Hcy and are also safe at higher doses. Studies have suggested that lowering Hcy with low‐dose folate and cyanocobalamin, or better yet, incorporating L‐methylfolate and methylcobalamin, makes these subgroups of patients more effective and safer to treat.

In addition to the fact that IS is a heterogeneous disease due to multiple pathologic mechanisms, which may mask the preventive effects of folate and vitamin therapy on IS, the mechanisms described above partially explain why Hcy‐reducing clinical trials (e.g., the VISP, HOPE‐2, and VITATOPS trials) have failed to report any improvement in cardiovascular disease risk and clinical outcomes. Perhaps in addition to vitamin supplementation, more effective ways to target Hcy metabolism should be investigated. Focus on improving renal clearance of Hcy in patients with renal insufficiency or switching to better L‐methylfolate and methylcobalamin.

In addition, the detection of MTHFR variants in stroke patients with elevated Hcy may help guide secondary prevention of stroke through adequate vitamin supplementation (Chen et al. 2023). If Hcy levels are elevated due to the presence of the MTHFR 677 TT genotype, treatment with oral 5‐MTHF should be considered; supplementation with 5‐MTHF reduces the negative effects of unmetabolized folate in the brain compared to folate, as the drug does not require any conversion by MTHFR (Bailey and Ayling 2018). This is because high levels of folate have the potential to mask symptoms of VB12 deficiency, which is common in older patients (Zhang et al. 2016). A study by Jadavji et al. (2019) suggests that dietary supplementation with 5‐MTHF, VB12, and choline may help alleviate some of the symptoms associated with IS, especially in elderly patients and individuals with the MTHFR C677T polymorphism. CBS deficiency can also lead to HHcy, and there are currently three approaches to treating CBS deficiency: patients who respond to vitamins may be treated with VB6 (50–250 mg/day) in combination with folate (0.4–5 mg/day) and/or VB12 (0.02–1 mg/day), and those who are vitamin non‐responders may be treated with a diet that restricts Met intake and is supplemented with cystine; VB6, folate, and VB12 are cofactors in Met metabolism, so VB6 non‐responders may need to be treated with VB6, folate, and VB12. Betaine, a methyl donor that remethylates Hcy to Met, has also been used as an adjuvant therapy (Yap 2003).

In addition to traditional vitamin supplements, other methods targeting Hcy metabolism were also explored. In a genetic model of CBS‐deficient mice, experiments by Gu et al. (2020) confirmed that Cbs^+/−^ mice showed elevated plasma Hcy and that elevated plasma Hcy exacerbated cerebral vascular injury, whereas administration of the NMDA receptor antagonist, memantine, protected Cbs^+/−^ mice from stroke and had a protective effect on the BBB. This experiment demonstrates that elevated plasma Hcy exacerbates cerebrovascular injury and suggests that NMDA antagonists may be a strategy to prevent reperfusion injury after acute IS in patients with mild HHcy. Taurine is a sulfur‐containing amino acid and is a product of cysteine in the trans‐sulfur pathway of homocysteine. New evidence from a phase 1/phase 2 clinical trial suggests that taurine may be a promising option for the treatment of vascular complications associated with homocystinuria because it improves otherwise diminished endothelial function in patients with CBS deficiency (Van Hove et al. 2019). Betaine, also known as trimethylglycine, is a natural methyl donor that can provide methyl groups to re‐methylate homocysteine into methionine, thereby reducing homocysteine levels. Betaine has functions such as regulating lipid metabolism and protecting vascular endothelial cells, and it has certain benefits for the cardiovascular system. The research results of Dai et al. (2022) indicate that supplementing betaine can reduce the level of SAH in the plasma and prevent atherosclerosis caused by SAHH deficiency by inhibiting inflammation, inhibiting the proliferation and migration of smooth muscle cells. Liu et al. (2017) conducted research on the anti‐atherosclerotic effect of allicin and tested the impact of allicin treatment on the plasma homocysteine levels of patients with coronary heart disease who had hyperhomocysteinemia. The research found that allicin treatment could reduce the serum homocysteine levels in HHcy rats and improve their damaged endothelial function. Moreover, allicin significantly decreased total cholesterol and triglycerides, indicating that it has the potential to prevent atherosclerosis by reducing Hcy and improving lipid metabolism. Hyperhomocysteinemia can induce oxidative stress, increase the production of reactive oxygen species, and damage vascular endothelial cells and nerve cells. N‐acetylcysteine (NAC) has antioxidant properties, which can increase the synthesis of glutathione within cells and alleviate oxidative stress damage; at the same time, NAC can also act as a precursor of cysteine, increasing the content of cysteine in the body and thereby promoting the metabolism of homocysteine and reducing its level (Aykin‐Burns et al. 2005). In the study by Kasperczyk et al. (2016), NAC has been shown to have a reducing effect on the homocysteine levels of patients exposed to lead. Traditional Chinese medicine has unique therapeutic effects and advantages in the treatment of ischemic stroke related to homocysteine. Its treatment model is based on syndrome differentiation and disease differentiation and is carried out based on regulating the internal organs and maintaining a balance of yin and yang. This therapy not only can reduce the concentration of homocysteine in the blood serum, increase the treatment rate, control rate, and cure rate of ischemic stroke, but also shows obvious superiority in improving the quality of life of patients, reducing the recurrence rate and the incidence of endpoint events (Ning et al. 2024).

In conclusion, in high‐risk populations, screening for Hcy levels and their risk factors and developing individualized prevention and treatment protocols for appropriate interventions based on an individual's Hcy levels and associated other risk factors may help to reduce the risk of IS.

## 6 Summary

IS has caused great harm worldwide, and epidemiologic studies have shown that HHcy is an independent risk factor for IS. Cofactor nutritional disorders and/or mutations in key enzyme genes can affect Hcy metabolic processes, leading to HHcy; elevated Hcy triggers a series of pathological processes, such as cerebrovascular injury, cerebral thrombosis, and reduced cerebral blood flow, through mechanisms related to inflammation and oxidative stress, neurotoxicity, and epigenetic dysregulation, which can lead to the onset and progression of IS. However, the specific pathogenesis of HHcy‐induced IS remains unclear. At this stage, HHcy‐associated IS is mainly prevented and treated with vitamin supplementation therapy; however, whether Hcy‐lowering therapy with vitamin supplementation has the potential to improve the clinical prognosis of patients with HHcy‐associated IS is unclear, and several clinical trials in which vitamin supplementation has lowered plasma Hcy concentrations have failed to reach a consistent conclusion. It is worth noting that non‐vitamin therapies targeting oxidative stress and endothelial dysfunction represent a promising treatment option. Such therapies may be more effective in counteracting vascular damage associated with HHcy.

## 7 Future Directions

Overall, Hcy has shown its potential as a risk factor in IS studies. However, it is specific mechanisms and clinical applications still require further research and validation to clarify the causal relationship of these correlations to discover more therapeutic targets for interventions in the treatment and prevention of HHcy‐related IS patients. The interrelationship between vitamin cofactors and Hcy metabolism is complex; genetic abnormalities and nutritional deficiencies can only partially explain HHcy, and hormonal and metabolic factors, as well as multivitamin supplementation therapy, maybe a clue to correcting Hcy levels in patients; therefore, it is important to find strategies to reduce Hcy levels. Although the available data suggest that vitamin supplementation therapy holds great promise for the prevention and treatment of HHcy‐related IS, a great deal of work remains to be done to determine the critical window of vitamin supplementation's therapeutic efficacy and identify the patients most likely to benefit. Additional large‐scale clinical trials evaluating the effects of vitamin supplementation therapy on IS risk in populations are necessary to clarify whether vitamin supplementation therapy has the potential to significantly reduce IS risk in HHcy populations, as well as to determine the optimal dosage of vitamin supplementation and further mechanistic studies are needed to investigate the dynamic and complex interactions of vitamin supplementation in altering Hcy metabolism. Although vitamin supplements remain the cornerstone of HHcy management, inconsistent cardiovascular outcomes suggest the need for a more comprehensive treatment approach. The future of HHcy treatment may go beyond vitamin supplements, including drugs targeting cysteine and prevention of the pathways related to HHcy‐induced ischemic stroke. Future research should focus on addressing the multifactorial nature of Hcy metabolism and its impact on ischemic stroke by improving treatment strategies, identifying appropriate patient subgroups, and exploring new non‐vitamin therapies.

## Author Contributions

**Bin Li:** conceptualization (lead), methodology (lead), writing – original draft (lead). **Yushun Kou:** methodology (equal), visualization (equal), writing – review and editing (equal). **Lingna Zhang:** methodology (supporting), visualization (lead). **Lin Yi:** conceptualization (equal), funding acquisition (lead), methodology (equal), supervision (lead).

## Ethics Statement

The authors have nothing to report.

## Consent

The authors have nothing to report.

## Conflicts of Interest

The authors declare no conflicts of interest.

## Data Availability

The datasets used and/or analyzed during the current study are available from the corresponding author upon reasonable request.
